# An Efficient Implementation Method for Distributed Fusion in Sensor Networks Based on CPHD Filters

**DOI:** 10.3390/s24010117

**Published:** 2023-12-25

**Authors:** Liu Wang, Guifen Chen

**Affiliations:** School of Electronic and Information Engineering, Changchun University of Science and Technology, Changchun 130022, China; wangl95@ccu.edu.cn

**Keywords:** distributed fusion, DG-CPHD, parallel inverse covariance intersection, wave filter

## Abstract

A highly efficient implementation method for distributed fusion in sensor networks based on CPHD filters is proposed to address the issues of unknown cross-covariance fusion estimation and long fusion times in multi-sensor distributed fusion. This method can effectively and efficiently fuse multi-node information in multi-target tracking applications. Discrete gamma cardinalized probability hypothesis density (DG-CPHD) can effectively reduce the computational burden while ensuring computational accuracy similar to that of CPHD filters. Parallel inverse covariance intersection (PICI) can effectively avoid solving high-dimensional weight coefficient convex optimization problems, reduce the computational burden, and efficiently implement filtering fusion strategies. The effectiveness of the algorithm is demonstrated through simulation results, which indicate that PICI-GM-DG-CPHD can substantially reduce the computational time compared to other algorithms and is more suitable for distributed sensor fusion.

## 1. Introduction

**Figure 1:**
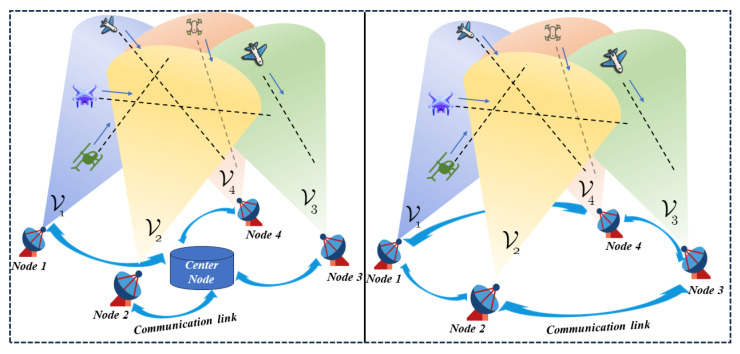
Schematic diagram of centralized sensor network and distributed sensor network.

The fusion estimation of multiple sensors can be divided into two types, centralized fusion and distributed fusion, as shown in Figure 1 [1]. The robustness and flexibility of distributed fusion offer several advantages. Distributed fusion sensors have a low data communication link bandwidth, allowing the efficient transmission of information. Additionally, the scalability of these sensors is excellent, making them adaptable to varying needs. Moreover, distributed fusion enables the acquisition of an optimal fusion algorithm when the correlation conditions are known. It is widely used in multi-sensor and multi-target tracking [2,3,4,5,6,7]. Scholars have conducted in-depth research on multi-sensor distributed fusion strategies. For example, Yu [8] suggested a method for distributed fusion filtering based on dynamic communication weights to tackle the issue of fusion errors in incomplete node information within distributed sensor networks. The proposed method aims to improve the situation by optimizing the fusion process in a finite time manner. Senel [9] conducted research and analysis in the fields of multi-sensor data fusion and real-time multi-target tracking. Liu [10] proposed a distributed sequential ellipsoidal intersection fusion estimation algorithm. This algorithm combines the local filter estimates obtained at the fusion center in order to achieve more precise fusion localization results. Additionally, it achieves this objective with reduced computational costs. Su [11] proposed an improved adaptive birth model based on pre-segmentation measurement in his research, which solves the problems of measurement origin uncertainty and cardinality overestimation in the distributed fusion of sensor networks. Pal [12] proposed a distributed fusion underwater wireless sensor network using local tracker optimal quantization estimation in his research.

**Figure 2:**
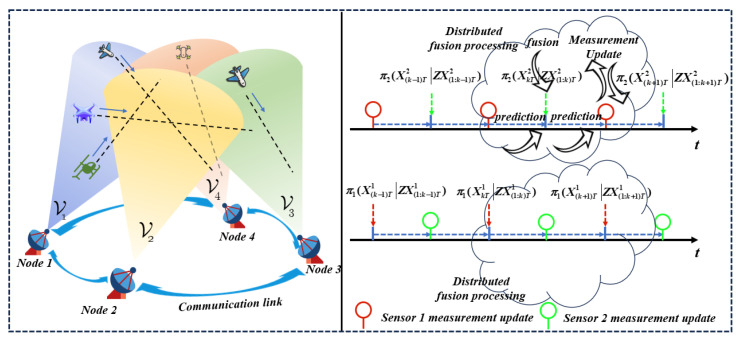
Distributed fusion processing of sensor networks.

In traditional distributed sensor networks, it can be assumed that nodes 1,2,⋯,l are synchronized and the common sampling period is *T*. $\pi_{a}(X_{kT}^{a}\left|ZX_{(1:k)T}^{a}\right.)$, a∈l, represents the posterior test of the sensor. Node 1 needs to send the posterior of the sensor to the next node (node 2) before fusion, as shown in Figure 2. However, in the actual process of distributed fusion in sensor networks, the accuracy of fusion is affected by factors such as the startup time, sampling period, and communication delay. Therefore, it is necessary to align the time and then fuse the predicted posterior set based on fusion criteria. A covariance intersection fusion (CI fusion) algorithm is proposed to address issues related to multi-sensor fusion estimation with unknown cross-covariance. CI fusion has the advantage of being able to combine covariance without taking into account the correlation between nodes [13,14,15,16,17,18], allowing for a conservative estimation. Scholars have conducted in-depth research on covariance cross-fusion. For example, Sun [19] proposed a distributed estimation approach utilizing inverse covariance intersection. This algorithm was proven to be useful in efficiently addressing challenges related to cross-correlation and dynamic-state estimation in wireless sensor networks. Liu [20] proposed sequential inverse covariance intersection (SICI) fusion Kalman filtering, which avoids the calculation of the cross-covariance between local filters and is less conservative than the previous sequential covariance intersection (SCI) filtering; Sun [21] introduced an algorithm that utilizes inverse covariance intersection (ICI) technology for distributed iterative diffusion estimation. The aim is to address correlation problems with unknown solutions and achieve accurate uncertainty estimates similar to the true mean square error. Hu [22] proposed, in his research, that, by analogy with batch CI fusion, hierarchical covariance intersection (HCI) fusion independent of the hierarchical structure and SCI fusion independent of the fusion order can be proposed, which can avoid the potential negative impact of uncertainty in the fusion process. Wang B [23] introduced a distributed multi-target tracking algorithm using a multi-Bernoulli (MB) filter that is based on the generalized covariance intersection (GCI). Wang L [24] proposed a method in his research that utilizes the inverse covariance cross-fusion algorithm to fuse the signals of various subsystems, solving the convex optimization problem of high-dimensional weight coefficients, reducing the burden of data computation, and minimizing the data fusion time. Park [25] proposed the inverse covariance intersection (ICI) in his research, extending it to multi-sensor and multi-objective tracking systems. However, the above works mainly focus on the research and analysis of the fusion strategy, without considering its efficient implementation. Although the fusion criteria can improve the operation accuracy of the system to a certain extent, doing so may reduce the operation efficiency. The main reasons can be summarized as follows: the complexity of the filtering methods increases with the number of targets, the complexity of the filtering methods increases exponentially with the operation time, the high-dimensional weight coefficient convex optimization problem of the fusion criteria themselves occurs, the reuse of fusion criteria increases the operation time, and the uncertainty caused by the hierarchy and order in the fusion process will affect the efficiency of the algorithm to a certain extent.

Scholars have conducted in-depth research and analysis on the efficient implementation of multi-sensor fusion strategies. For example, Li [26] proposed a real-time fusion algorithm for asynchronous sensor systems and a real-time sequential (RTS) fusion method, RTS-GCI, based on generalized covariance intersection (GCI) to achieve fast sensor filtering. The implementation of distributed fusion based on maximum probability association (MPA-DF), proposed by Li [27,28], and the generalized covariance intersection (GCI) distributed fusion method (referred to as GCI-DF) can achieve efficient filtering to a certain extent. In the above research, by improving the fusion strategy and adjusting the level and order of the fusion process to reduce the computational burden, the operational efficiency of the algorithm could be improved to a certain extent. Therefore, it can be seen that the efficient implementation of a fusion strategy applied to a multi-sensor multi-target system is one of the future development directions of multi-sensor distributed fusion multi-target tracking.

This article proposes an efficient implementation method for the distributed fusion of sensor networks based on cardinalized probability hypothesis density (CPHD) filters, which addresses the issues of unknown cross-covariance fusion estimation and long fusion times in multi-sensor distributed fusion. The parallel inverse covariance intersection (PICI) fusion algorithm is applied in local estimation fusion, effectively addressing the issue of the convex optimization of weight coefficients with a high number of sensors in multi-sensor distributed fusion. Additionally, the PICI fusion algorithm is employed in discrete gamma cardinalized probability hypothesis density (DG-CPHD) and Gaussian mixture discrete gamma cardinalized probability hypothesis density (GM-DG-CPHD), successfully reducing the running time. The simulation results demonstrate the efficacy of the algorithm.

## 2. Research Background

### 2.1. GM-CPHD Filter

The multi-objective density function f(X) is a real-valued function $RFSX=\{x_{1},\cdots ,x_{n}\}$, and the multi-objective density function is a characteristic of a random finite set (RFS). The set integral of the multi-objective density function is defined as

(1) $$\int f(X)\delta X≜f(\phi )+\sum_{n=1}^{\infty }\frac{1}{n!}\int f(\{x_{1},\cdots ,x_{n}\})dx_{1}\cdots dx_{n}$$

where X is the multi-target set and *n* is the target quantity.

The probability-generating function (PGF) G[h] and probability hypothesis density D(x) are defined as

(2) $$G[h]≜\int f(X)\prod_{x\in X}h(x)\delta X$$

(3) $$D(x)≜\frac{\delta }{\delta x}G[h]\left|{}_{h=1}\right.$$

#### 2.1.1. CPHD Filter

The CPHD filter [29,30,31] merges the propagation intensity function and cardinality distribution (which represents the probability distribution of the target number) by considering the clutter RFS as an independent and identically distributed (i.i.d.) process.

**Assumption** **1.**  *Both prior and posterior multi-objective random finite sets are i.i.d. clustering processes*.

**Assumption** **2.**  *Each target moves independently, and a single target undergoes a Markov transition density* $p_{t,k\left|k-1\right.}(x\left|x^{\prime }\right.)$

**Assumption** **3.**  *Newly born targets can independently appear in sports scenarios. Each target moves independently, and a single target undergoes Markov transfer density* $p_{t,k\left|k-1\right.}(x\left|x^{\prime }\right.)$. *The density of the newborn target is* $b_{t,k\left|k-1\right.}(X)$, *the strength function of the random finite set is expressed as* D(x), *and the PGFL is* G[h].

**Assumption** **4.**  *The sensor detects a single target with state x as* $p_{d,k}(x)$ *at time step k with probability* $p_{d}$.

The CPHD filter’s definition is based on a multi-object distribution and encompasses processes that are independent and identically distributed. If we assume that the cardinality distribution p(n) of the point process is $\left|X\right|=n$, then the PGFL G[h] and the probability assumption density D(x) of CPHD can be stated as

(4) $$f(X)≜n!\;\cdot \;p(n)\cdot f(x_{1})\cdots f(x_{n})$$

(5) $$G[h]=\sum_{n=0}^{\infty }p(n){\left(\int h(u)\cdot f(u)du\right)}^{n}$$

(6) $$D(x)≜\frac{\delta }{\delta x}G[h]\left|{}_{h=1}\right.=f(x)\sum_{n=1}^{\infty }n\cdot p(n)$$

1. Prediction of CPHD

(7) $$p_{k,t\left|t-1\right.}(n)=\sum_{j=0}^{n}p_{b}(n-j)\sum_{h=j}^{\infty }\left(\begin{array}{c} h\\ j \end{array}\right)p_{k,t}^{j}{\left(1-p_{k,t}\right)}^{h-j}p_{t-1\left|t-1\right.}(h)$$

(8) $$D_{k\left|k-1\right.}(x)=D_{b}(x)+\int {}_{X}p_{s}(x^{\prime })p_{t}(x\left|x^{\prime }\right.)D_{k\left|k-1\right.}(x^{\prime })dx^{\prime }$$

(9) $${\hat{N}}_{k\left|k-1\right.}={\hat{N}}_{a,k}+{\hat{N}}_{b,k}$$

Here, $p_{s}(x^{\prime })$ is a known objective transition function with a previous state; $p_{t}(x\left|x^{\prime }\right.)$ is the survival probability of the target with a previous state; ${\hat{N}}_{a,k}$ is the expected number of new goals; and ${\hat{N}}_{b,k}$ is the expected number of goals that survive in time step *k* − 1.

2. Updates to CPHD

(10) $$p_{k,t\left|t\right.}(n)=\frac{L_{t}^{0}(d_{k,t\left|t-1\right.}(\cdot ),y_{t},n)p_{k,t\left|t-1\right.}(n)}{\sum_{i=0}^{\infty }L_{t}^{0}(d_{k,t\left|t-1\right.}(\cdot ),y_{t},i)p_{k,t\left|t-1\right.}(i)}$$

(11) $$d_{k,t\left|t\right.}(x)=L_{yt}(x)d_{k,t\left|t-1\right.}(x)$$

Here, 1 is the generalized likelihood function.

3. The complexity of CPHD

The complexity of PHD recursion is $O(mn_{\max})$, while the CPHD algorithm can be seen as evaluating the elementary pair production function m + 1 times, with complexity of $O(m^{3})∼O{\left|Z\right|}^{3}n_{\max})$, where m is the number of evaluations and $n_{\max}$ is the maximum number of targets. When $n_{\max}>m$, CPHD can be considered as $\left(m^{3}+n_{\max}m^{2}-\frac{m^{2}}{2})∼O(n_{\max}m^{2}\right)$.

#### 2.1.2. GM-CPHD Filter

Based on the above conditions, the recursive form of GM-CPHD can be given as follows.

1. Prediction of GM-CPHD

If we make the assumption that the posterior intensity at time *k* − 1 follows a Gaussian mixture in the given format, then

(12) $$D_{k\left|k-1\right.}^{\prime }(x)=\sum_{i=1}^{J_{k-1}}\omega_{k-1}^{i}N(x;m_{k-1}^{i},P_{k-1}^{i})$$

The predicted *k*-time intensity is also a Gaussian mixture, given by the following equation:

(13) $$D_{k\left|k-1\right.}^{\prime }(x)=d=D_{s,k\left|k-1\right.}^{\prime }(x)+\gamma_{k}(x)$$

(14) $$D_{s,k\left|k-1\right.}^{\prime }(x)=p_{s,k}\sum_{j=1}^{J_{k-1}}\omega_{k-1}^{j}N(x;m_{k-1}^{j},P_{k-1}^{j})$$

(15) $$m_{s,k\left|k-1\right.}^{j}=F_{k-1}m_{k-1}^{j}$$

(16) $$P_{s,k\left|k-1\right.}^{j}=Q_{k-1}+F_{k-1}P_{k-1}^{j}F_{k-1}^{Τ}$$

(17) $$D_{\beta ,k\left|k-1\right.}^{\prime }(x)=\sum_{j=1}^{J_{k-1}}\sum_{l=1}^{J_{\beta ,k}}\omega_{k-1}^{j}\omega_{\beta ,k}^{l}N(x;m_{\beta ,k\left|k-1\right.}^{j,l},P_{\beta ,k\left|k-1\right.}^{j,l})$$

(18) $$m_{\beta ,k\left|k-1\right.}^{j,l}=F_{\beta ,k-1}^{l}m_{k-1}^{j}+d_{\beta ,k-1}^{l}$$

(19) $$P_{\beta ,k\left|k-1\right.}^{j,l}=Q_{\beta ,k-1}^{l}+F_{\beta ,k-1}^{l}P_{\beta ,k-1}^{j}{\left(F_{\beta ,k-1}^{l}\right)}^{Τ}$$

2. Update for GM-CPHD

Assuming that the predicted strength has a Gaussian mixture form, it is

(20) $$D_{k\left|k-1\right.}^{\prime }(x)=\sum_{i=1}^{J_{k\left|k-1\right.}}\omega_{k\left|k-1\right.}^{i}N(x;m_{k\left|k-1\right.}^{i},P_{k\left|k-1\right.}^{i})$$

The predicted *k*-time intensity is also a Gaussian mixture, given by the following equation:

(21) $$D_{k}^{\prime }(x)=(1+p_{d,k})D_{k\left|k-1\right.}^{\prime }(x)+\sum_{z\in Z_{k}}D_{d,k}(x;z)$$

At this point,

(22) $$D_{d,k}^{\prime }(x;z)=\sum_{j=1}^{J_{k\left|k-1\right.}}\omega_{k}^{j}N(x;m_{k\left|k\right.}^{j},P_{k\left|k\right.}^{j})$$

(23) $$\omega_{k}^{j}(z)=\frac{p_{d,k}\omega_{k\left|k-1\right.}^{j}q_{k}^{j}(z)}{\kappa_{k}(z)+p_{d,k}\sum_{l=1}^{J_{k\left|k-1\right.}}w_{k\left|k-1\right.}^{l}q_{k}^{l}(z)}$$

(24) $$m_{k\left|k\right.}^{j}(z)=m_{k\left|k-1\right.}^{j}+K_{k}^{j}(z-H_{k}m_{k\left|k-1\right.}^{j})$$

(25) $$P_{k\left|k\right.}^{j}=[I-K_{k}^{j}H_{k}]P_{k\left|k-1\right.}^{j}$$

(26) $$K_{k}^{j}=P_{k\left|k-1\right.}^{j}H_{k}^{T}{\left(H_{k}P_{k\left|k-1\right.}^{j}H_{k}^{T}+R_{k}\right)}^{-1}$$

3. The complexity of GM-CPHD

At this point, the complexity of CPHD recursion is $O(m^{3})∼O{\left|Z\right|}^{3}n_{\max})$, and the complexity of GM-CPHD will increase significantly as the number of targets increases.

### 2.2. Discrete Gamma CPHD Filter

#### 2.2.1. DG-CPHD Filter

1. Prediction of DG-CPHD

Flávio [32,33] aimed, in his research, to model the cardinality distribution in CPHD filtering as a discrete gamma distribution, while ensuring cardinality accuracy and variance, reducing the complexity of CPHD filtering and achieving fast filtering. The DG-CPHD filtering process assumes that the states of multiple objectives follow a clustering process that is both independent and identically distributed. The distribution of cardinality is modeled using a discrete gamma distribution. The unique characteristic of this discretized gamma distribution is that it enables straightforward calculations for the approximation of the first-order and second-order matrices of the posterior cardinality distribution. This approximation method effectively bypasses the limitations imposed by binomial filters.

The assumptions for the discrete gamma CPHD filter are

(27) $$p_{\Xi }(X)=dGamma_{\alpha ,\beta }(n)\cdot n!\prod_{i=1}^{n}p(x_{i})$$

where 1 is the spatial probability density of each object.

The predicted intensity function is

(28) $$D_{k\left|k-1\right.}^{\prime \prime }(x)=D_{b}^{\prime \prime }(x)+\int {}_{X}p_{s}(x^{\prime })p_{t}(x\left|x^{\prime }\right.)D_{k\left|k-1\right.}^{\prime \prime }(x^{\prime })dx^{\prime }$$

The parameters of the posterior discrete gamma cardinality distribution at time *k* − 1, with a ratio of $\alpha_{k-1}$ (shape) and $\beta_{k-1}$ (rate), are

(29) $$\alpha_{k\left|k-1\right.}=\frac{\mu_{N,k\left|k-1\right.}^{2}}{\sigma_{N,k\left|k-1\right.}^{2}}$$

(30) $$\beta_{k\left|k-1\right.}=\frac{\mu_{N,k\left|k-1\right.}}{\sigma_{N,k\left|k-1\right.}^{2}}$$

(31) $${\hat{N}}_{k\left|k-1\right.}={\hat{N}}_{a,k}+{\hat{N}}_{b,k}=\mu_{N,k\left|k-1\right.}$$

(32) $$\sigma_{N,k\left|k-1\right.}^{2}={\hat{N}}_{a,k}+{\hat{N}}_{b,k}+{\langle p_{s},\varsigma \rangle }^{2}\alpha_{k-1}\beta_{k-1}^{-1}(\beta_{k-1}^{-1}-1)$$

The primary distinction lies in how the estimated distribution of cardinality is approximated. In the case of the DG-CPHD filter, this approximation is represented by the discrete gamma cardinality parameters $\alpha_{k-1}$ and $\beta_{k-1}$.

2. Update of DG-CPHD

(33) $$D_{k\left|k-1\right.}^{\prime \prime }(x)=\frac{p_{d}(x)}{\mu_{N,k\left|k+1\right.}}\sum_{z\in Z_{k}}\frac{l_{d}(x)}{\lambda c(z)}\Theta_{k}[Z_{k}\backslash \left\{z\right\}]D_{k\left|k-1\right.}^{\prime \prime }(x)+\frac{p_{d}(x)}{\mu_{N,k\left|k+1\right.}}\Theta_{k}[Z_{k}]D_{k\left|k-1\right.}^{\prime \prime }(x)$$

(34) $$\alpha_{k\left|k-1\right.}=\frac{\mu_{N,k}^{2}}{\sigma_{N,k}^{2}}$$

(35) $$\beta_{k\left|k-1\right.}=\frac{\mu_{N,k}}{\sigma_{N,k}^{2}}$$

Define $\mu_{N,k\left|k-1\right.}$ as $\alpha_{k\left|k-1\right.}\beta_{{}_{k\left|k-1\right.}}^{-1}$

(36) $$\mu_{N,k}=\theta_{1,0}+\theta_{0,1}\cdot \langle p_{d},\varsigma \rangle$$

(37) $$\sigma_{N,k}^{2}=\theta_{2,0}-\theta_{1,0}+2\theta_{1,1}\cdot \langle p_{d},\varsigma \rangle +\theta_{0,2}\cdot {\langle p_{d},\varsigma \rangle }^{2}-(\mu_{N,k}^{2}-\mu_{N,k})$$

(38) $$\Theta_{k}[Z]≜\frac{\sum_{j=0}^{\left|Z\right|}{\hat{G}}^{j+1}(\langle q_{d},\varsigma \rangle )\sigma_{m=\left|Z\right|,j}(\frac{\langle q_{d}l_{z1},\varsigma \rangle }{\lambda c(z_{1})},\cdots ,\frac{\langle q_{d}l_{zm},\varsigma \rangle }{\lambda c(z_{m})})}{\sum_{i=0}^{m_{k}}{\hat{G}}^{i}(\langle q_{d},\varsigma \rangle )\sigma_{m=\left|Z_{k}\right|,i}(\frac{\langle q_{d}l_{zk1},\varsigma \rangle }{\lambda c(z_{1k})},\cdots ,\frac{\langle q_{d}l_{zkm},\varsigma \rangle }{\lambda c(z_{km})})}$$

(39) $$\theta_{u,v}≜\frac{\sum_{j=0}^{m_{k}}j^{u}{\hat{G}}^{j+v}(\langle q_{d},\varsigma \rangle )\sigma_{m=\left|Z_{k}\right|,i}(\frac{\langle q_{d}l_{zk1},\varsigma \rangle }{\lambda c(z_{1k})},\cdots ,\frac{\langle q_{d}l_{zkm},\varsigma \rangle }{\lambda c(z_{km})})}{\sum_{i=0}^{m_{k}}{\hat{G}}^{i}(\langle q_{d},\varsigma \rangle )\sigma_{m=\left|Z_{k}\right|,j}(\frac{\langle q_{d}l_{zk1},\varsigma \rangle }{\lambda c(z_{1k})},\cdots ,\frac{\langle q_{d}l_{zkm},\varsigma \rangle }{\lambda c(z_{km})})}$$

The primary distinction lies in how the estimated distribution of cardinality is approximated. In the case of the DG-CPHD filter, this approximation is represented by the discrete gamma cardinality parameters $\alpha_{k-1}$ and $\beta_{k-1}$.

3. The complexity of DG-CPHD

The computational complexity of CPHD is  $O(m^{3}n\max)$, and the DG-CPHD filter is constrained to using a finite number of terms, ${\bar{n}}_{\max}$, to approximate derivatives of the probability-generating function (PGF) G[h]. The results demonstrate that ${\bar{n}}_{\max}$ is not as prone to sensitivity to the number of target-generated measurements.

References [32,33] show that when the value of λ increases, the complexity of both the CPHD and DG-CPHD filters is primarily influenced by the total number of measurements. This means that as the number of false alarms increases, the running times of these filters increase at a sub-exponential rate, with a maximum limit of $O(m^{3})$ operations.

#### 2.2.2. GM-DG-CPHD Filter

1. Prediction of GM-DG-CPHD

Based on the above GM-CPHD filtering process, the GM-DG-CPHD prediction process can be written as

(40) $$D_{k\left|k-1\right.}^{\prime \prime \prime }(x)=\sum_{i=1}^{J_{k-1}}\omega_{k\left|k-1\right.}^{i}N(x;m_{k\left|k-1\right.}^{i},P_{k\left|k-1\right.}^{i})$$

$\alpha_{k\left|k-1\right.}\beta_{{}_{k\left|k-1\right.}}^{-1}$ are the same as above, but, at this point,

(41) $$\mu_{N,k\left|k-1\right.}=\sum_{i=1}^{I_{b,k}}\omega_{b,k}^{i}+\alpha_{k\left|k-1\right.}\beta_{{}_{k\left|k-1\right.}}^{-1}$$

(42) $$\sigma_{N,k\left|k-1\right.}^{2}=\mu_{N,k\left|k-1\right.}+p_{s}^{2}\alpha_{k\left|k-1\right.}\beta_{{}_{k\left|k-1\right.}}^{-1}(\beta_{{}_{k\left|k-1\right.}}^{-1}-1)$$

2. Update of GM-DG-CPHD

(43) $$D_{k\left|k-1\right.}^{\prime \prime \prime }(x)=\sum_{i=1}^{J_{k-1}}\omega_{k}^{i}N(x;m_{k}^{i},P_{k}^{i})$$

$\alpha_{k\left|k-1\right.}\beta_{{}_{k\left|k-1\right.}}^{-1}$ are the same as above, but, at this point,

(44) $$\mu_{N,k}=\theta_{1,0}+\theta_{0,1}\cdot (1-p_{d})$$

(45) $$\sigma_{N,k}^{2}=\theta_{2,0}-\theta_{1,0}+2\theta_{1,1}\cdot (1-p_{d})+\theta_{0,2}\cdot {\left(1-p_{d}\right)}^{2}-(\mu_{N,k}^{2}-\mu_{N,k})$$

(46) $$w_{u,k}^{j}=\frac{\left(1-p_{d})\Theta_{k}[Z_{k}\right]}{\alpha_{k\left|k-1\right.}\beta_{{}_{k\left|k-1\right.}}^{-1}}w_{u,k\left|k-1\right.}^{j}$$

(47) $$m_{u,k}^{j}=m_{k\left|k-1\right.}^{j}$$

(48) $$P_{u,k}^{j}=P_{k\left|k-1\right.}^{j}$$

3. The complexity of GM-DG-CPHD

Similarly, the complexity of GM-DG-CPHD will increase significantly as the number of targets increases.

### 2.3. GCI Fusion Strategy

In the fusion of distributed sensor networks, the GCI fusion algorithm is traditionally used. The core of the GCI fusion criterion is to minimize the weighted sum of the Kullback–Leibler divergence (KLD) relative to a given distribution set by fusing a posterior. Under the GCI fusion rule, the common product form of Bayesian rules is replaced by the exponential mixing of geometric means or distributions [34].

(49) $$\pi_{\omega }(X_{kT}\left|Z_{(1:k)T}^{1}\right.,Z_{(1:k)T}^{1})=\frac{\pi_{1}{\left(X_{kT}^{1}\left|Z_{(1:k)T}^{1}\right.\right)}^{\omega_{1}}\pi_{2}{\left(X_{kT}^{2}\left|Z_{(1:k)T}^{2}\right.\right)}^{\omega_{2}}}{\int \pi_{1}{\left(X_{kT}^{1}\left|Z_{(1:k)T}^{1}\right.\right)}^{\omega_{1}}\pi_{2}{\left(X_{kT}^{2}\left|Z_{(1:k)T}^{2}\right.\right)}^{\omega_{2}}\delta X}$$

(50) $$\pi_{\omega }=\arg\underset{\omega }{\min}(1-\omega )D(\pi \left|\pi_{1}\right.)+\omega D(\pi \left|\pi_{2}\right.)$$

Here, *D* is the Kullback–Leibler divergence, and $\omega_{1}+\omega_{2}=1$.

However, the disadvantage of the GCI fusion strategy is that it generates many Gaussian components after fusion, increasing the computational burden and complexity. There are also some Gaussian components with low weights that need to be pruned and merged in subsequent calculations.

## 3. A Fast Fusion Method for Discrete Gamma CPHD Real-Time Sequences

### 3.1. PICI Fusion Algorithm

**Figure 3:**
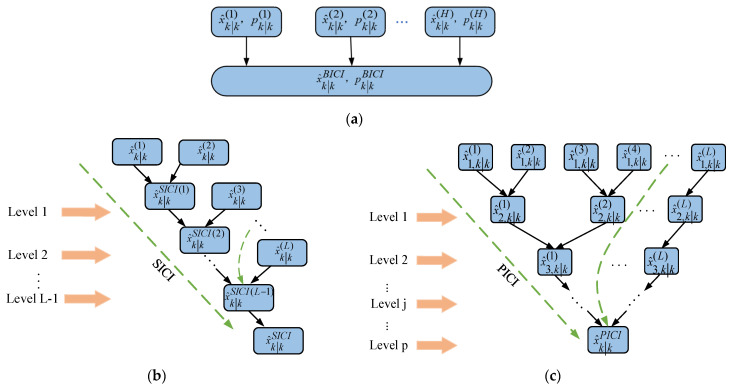
Common fusion strategies: (**a**) BICI integration strategy; (**b**) SICI integration strategy; (**c**) PICI integration strategy.

Common fusion methods include batch inverse covariance intersection (BICI), sequential inverse covariance intersection (SICI), and parallel inverse covariance intersection (PICI). As shown in Figure 3, BICI fusion enhances convex optimization problems with high-dimensional weight coefficients among the mentioned solutions. On the other hand, SICI fusion eliminates the need to solve high-dimensional weight coefficient convex optimization problems, but, when there are too many sensors, it will consume a lot of time due to multiple sequential fusion.

As the system incorporates multiple sensors, the SICI fusion algorithm necessitates the execution of L-1 pairwise fusions, which can be time-consuming. With a fixed number of sensors L, as the layers increase, the number of ICI fusion devices for the two sensors will progressively decrease in an exponential manner. Therefore, when L is particularly large, the PICI algorithm can save a lot of computational time. In order to reduce the computational time of the system, a parallel covariance intersection fusion algorithm is used for operation. For this purpose, this study uses the PICI fusion strategy to achieve the efficient implementation of distributed fusion in sensor networks.

In the PICI multi-sensor fusion algorithm, the fusion estimation of ${\hat{x}}_{k\left|k\right.}^{i}(i,i+1,\cdots ,L)$ at different levels is determined by the subsystem estimation ${\hat{x}}_{k\left|k,2i-1\right.}^{ICI,j-1},{\hat{x}}_{k\left|k,2i\right.}^{ICI,j-1}$ and subsystem variance $P_{ICI,2i-1}^{j-1},P_{ICI,2i}^{j-1}$ of the ICI fusion of two sensors in the previous layer. Therefore, the PICI fusion algorithm is

(51) $${\hat{x}}_{k\left|k,i\right.}^{ICI,j}=K_{k,i}^{j}{\hat{x}}_{k\left|k,2i-1\right.}^{ICI,j-1}+L_{k}^{j}{\hat{x}}_{k\left|k,2i\right.}^{ICI,j-1}$$

(52) $$P_{ICI}^{j}={\left[{\left(P_{ICI,2i-1}^{j-1}\right)}^{-1}+{\left(P_{ICI,2i}^{j-1}\right)}^{-1}-{\left(\omega_{i}^{j}P_{ICI,2i-1}^{j-1}+(1-\omega_{i}^{j})P_{ICI,2i}^{j-1}\right)}^{-1}\right]}^{-1}$$

(53) $$K_{K}^{j}=P_{ICI,i}^{j}{\left[{\left(P_{ICI,2i-1}^{j-1}\right)}^{-1}-\omega_{i}^{j}(\omega_{i}^{j}P_{ICI,2i-1}^{j-1})+(1-\omega_{i}^{j})P_{ICI,2i}^{j-1}\right)}^{-1}]$$

(54) Lk,ij=PICI,ij[(PICI,2ii−1)−1−(1−ω)ij(ωPijICI,2i−1i−1+(1−ω)ijPICI,2ii−1)−1]

where $i=1,2,\cdots ,L_{j},j=1,2,\cdots L_{p}$, and $L_{j},L_{p}$ represent the numbers of filters in the *j*th layer and the final number of layers, respectively.

For this reason, the initial value can be considered as

(55) $${\hat{x}}_{k\left|k,i\right.}^{ICI,1}={\hat{x}}_{k\left|k\right.}^{i},P_{ICI,i}^{1}=P_{k\left|k\right.}^{i},L_{1}=L$$

The performance indicators for the minimization of ω∈[0,1] are

(56) $$\min J==\underset{\omega_{1}\in [0,1]}{\min}tr\left\{{\left[{\left(P_{k\left|k\right.}^{1}\right)}^{-1}+{\left(P_{k\left|k\right.}^{2}\right)}^{-1}-(\omega_{1}P_{k\left|k\right.}^{1}+(1-\omega_{1}){\left(P_{k\left|k\right.}^{2}\right)}^{-1}\right]}^{-1}\right\}$$

(57) $$\min J=\underset{\omega_{{}_{i}}^{j}\in [0,1]}{\min}tr\left\{{\left[{\left(P_{ICI,2i-1}^{j-1}\right)}^{-1}+{\left(P_{ICI,2i}^{j-1}\right)}^{-1}-(\omega_{i}^{j}P_{ICI,2i+1}^{j-1}+(1-\omega_{i}^{j}){\left(P_{ICI,2i}^{j-1}\right)}^{-1}\right]}^{-1}\right\}$$

**Figure 4:**
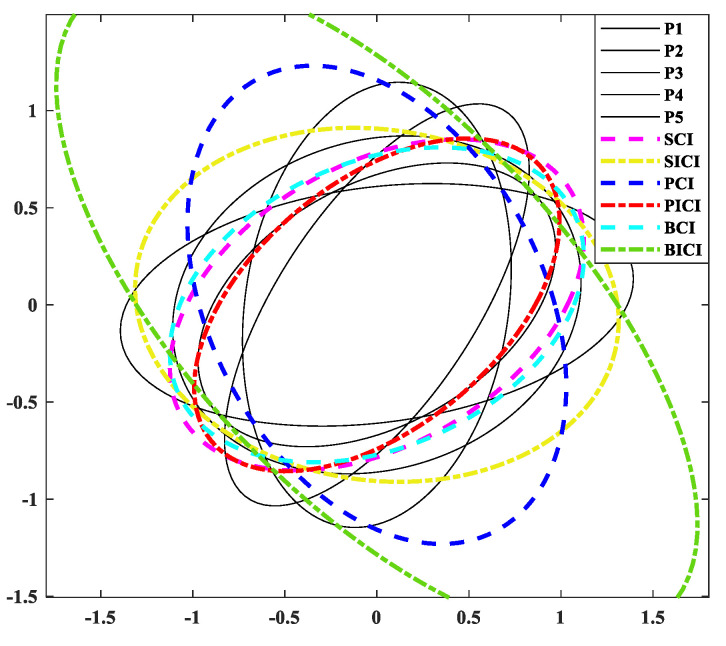
Comparison results of different covariance intersection fusion algorithms.

In order to validate the effectiveness of the PICI fusion algorithm, this study chose an L value of 5 and conducted a comparative analysis on the effects of SCI, SICI, PCI, PICI, BCI, and BICI through covariance ellipse validation. The results of the comparison of various covariance intersection fusion algorithms can be seen in Figure 4. From this, it can be seen that the PICI fusion algorithm has higher fusion accuracy than other algorithms.

### 3.2. Implementation of PICI-GM-DG-CPHD Algorithm

For the convenience of calculation, according to references [24,25], PICI is rewritten as

(58) $$x_{PICI}=K_{PICI}x_{A}^{j-1}+L_{PICI}x_{B}^{j-1}$$

(59) $$\begin{array}{ll} P_{PICI} & ={\left(P_{A}^{j-1}\right)}^{-1}+{\left(P_{B}^{j-1}\right)}^{-1}-(\omega_{PICI}P_{A}^{j-1}+(1-\omega_{PICI})P_{B}^{j-1})-1{)}^{-1}\\ & \;={\left({\left(P_{A,PICI}^{j-1}\right)}^{-1}+{\left(P_{B,PICI}^{j-1}\right)}^{-1}\right)}^{-1} \end{array}$$

From the above Equations (58) and (59), it can be seen that the fusion results of the PICI algorithm at the next level are determined by the fusion results of the previous level. To this end, PICI and ICI can be determined differently in terms of $P_{A}^{-1},P_{B}^{-1}$, and PICI needs to be divided into $P_{L_{P},k\left|k\right.}^{A(M)},P_{L_{P},k\left|k\right.}^{B(m)}$ types at different levels. When the number of sensors *L* is even, then $m=\frac{L}{2}$, and when the number of sensors *L* is odd, then $m=\frac{L-1}{2}$.

(60) $${\left(P_{A,ICI}^{\left(y\right)}\right)}^{-1}≜{\left(P_{A}^{\left(y\right)}\right)}^{-1}-\omega_{ICI}{\left(\omega_{ICI}P_{A}^{\left(y\right)}+(1-\omega_{ICI})P_{B}^{\left(y\right)}\right)}^{-1}$$

(61) $${\left(P_{B,ICI}^{y}\right)}^{-1}≜{\left(P_{B}^{y}\right)}^{-1}-\omega_{ICI}{\left(\omega_{ICI}P_{B}^{y}+(1-\omega_{ICI})P_{A}^{y}\right)}^{-1}$$

Here, y=1,2,⋯,m.

Considering the existence of multiple fusion scenarios in PICI, the results of different sensor combinations can be set as

(62) $${\hat{x}}_{\left(L\right)}^{PICI}=\sum_{i=1}^{L}M_{i}^{PICI}{\hat{x}}_{i}$$

(63) $$P_{\left(L\right)}^{PICI}=\sum_{i=1}^{L}N_{i}^{PICI}P_{i}$$

The weighting matrix is represented by

(64) $$M_{i}^{PICI}=M_{i(L)}^{PICI}\cdots M_{i(2)}^{PICI}M_{i(1)}^{PICI}$$

(65) $$N_{i}^{PICI}=N_{i(L)}^{PICI}\cdots N_{i(2)}^{PICI}N_{i(1)}^{PICI}$$

For this purpose, PICI-GM-CPHD can be represented as follows. As shown in Algorithm 1.
**Algorithm 1:** PICI-GM-CPHD filtering algorithm processPerform GM-DG-CPHD filtering processing:
  Input: $\left\{\omega_{k-1}^{i},m_{k-1}^{i},P_{k-1}^{i}\right\}$$\alpha_{k-1},\beta_{k-1}$$Z_{k}=\left\{z_{1},z_{2},\cdots ,z_{mk}\right\}$ **Prediction Step**
  for $i=1,2,\cdots I_{b,k}$ do   Define the correlation coefficients to predict the intensity of new students’ goals in Formula (40), and predict the intensity of new students’ goals   $I_{k\left|k-1\right.}:=I_{b,k}+I_{k-1}$
   for $i=I_{b,k}+1,\cdots ,I_{k\left|k-1\right.}$ do   Redefine the correlation coefficient to predict the strength of new targets in Formula (40) and predict the strength of surviving targets   Predict cardinality parameters based on Formulas (34), (35), (41), (42), (44), (45)   End for **Measurement Update Step**
  For $i=1,2,\cdots I_{k\left|k-1\right.}$ do   According to Formulas (43)–(48), update the intensity of newly detected targets and the prediction base parameters   Output:$\left\{\omega_{k}^{i},m_{k}^{i},P_{k}^{i}\right\}$$\alpha_{k},\beta_{k}$  End forFor l=1:L(*L* is number of sensors);   Calculate PICI fusion weight using covariance intersection;   Replace the covariance of the probability density a of $P_{A}^{y},P_{B}^{y}$ single sensor with   $\left\{\begin{array}{l} P_{A,ICI}^{y}=P_{A}^{y}+\frac{\omega_{ICI}}{1-\omega_{ICI}}P_{A}^{y}{\left(P_{B}^{y}\right)}^{-1}P_{A}^{y}\\ P_{B,ICI}^{y}=P_{B}^{y}+\frac{\omega_{ICI}}{1-\omega_{ICI}}P_{B}^{y}{\left(P_{A}^{y}\right)}^{-1}P_{B}^{y} \end{array}\right.$  Different calculations $P_{L_{P},k\left|k\right.}^{A(m)},P_{L_{P},k\left|k\right.}^{B(m)}$;   Calculate the next level’s fusion result based on the previous level’s fusion result;   Determine the PICI fusion results of multiple sensors based on Equations (64) and (65);   Calculate different PICI-GM-CPHD weighting matrices End for Estimate extraction.

## 4. Modeling and Simulation

To verify the effectiveness of the algorithm, this study applies it to the constant velocity (CV) model for validation. The linear model CV model is $CV(x)={\left(x\;\vartheta \;y\;v\right)}^{⊤}$, where the four state variables are x as the horizontal axis, ϑ as the angle with the x-axis (counterclockwise as positive), y as the vertical axis, and v as the linear velocity.

(66) $$F_{k}=\left[\begin{array}{cc} I_{2} & \Delta I_{2}\\ O_{2} & I_{2} \end{array}\right]$$

(67) $$Q_{K}=\sigma_{v}^{2}\left[\begin{array}{cc} \frac{\Delta^{4}}{4}I_{2} & \frac{\Delta^{3}}{2}I_{2}\\ \frac{\Delta^{3}}{2}I_{2} & \Delta^{2}I_{2} \end{array}\right]$$

(68) $$H_{k}=[\begin{array}{cc} I_{2} & O_{2} \end{array}],\;R_{k}=\sigma_{\varepsilon }^{2}I_{2}$$

We configure the GM-DG-CPHD filter for a sensor with a detection probability of 0.9 and a survival probability of 0.99. The uniform clutter of the moving target has a Poisson average rate of 5, and the birth density is located at (±1000 m, ±1000 m). All simulations are conducted using 120 Monte Carlo experiments, with the GM-DG-CPHD parameters set as follows: truncation threshold $T=10^{-5}$, merging threshold U=2, and maximum allowable number of Gaussian terms $J_{\max}=100$. The tracking performance of the moving targets is evaluated using the optimal sub-pattern assignment (OSPA) distance.

### 4.1. Comparison Results of Multiple Filtering Methods

**Figure 5:**
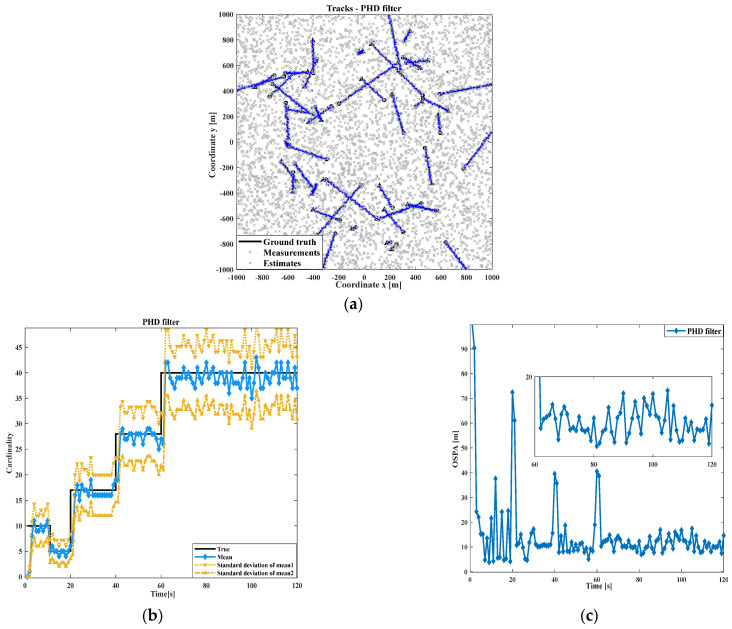
Comparison of PHD filtering results: (**a**) target tracking path result; (**b**) cardinal distribution results; (**c**) OSPA error results.

**Figure 6:**
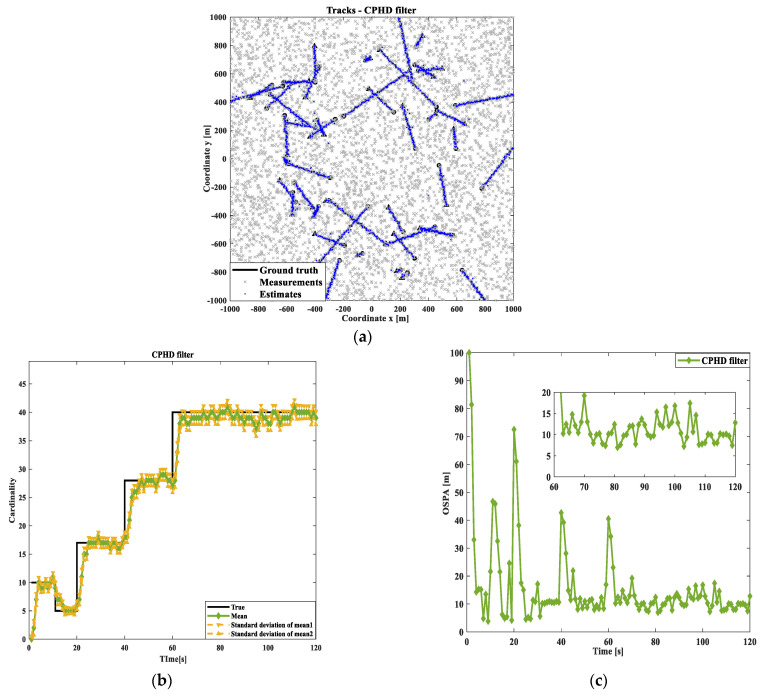
Comparison of CPHD filtering results: (**a**) target tracking path result; (**b**) cardinal distribution results; (**c**) OSPA error results.

**Figure 7:**
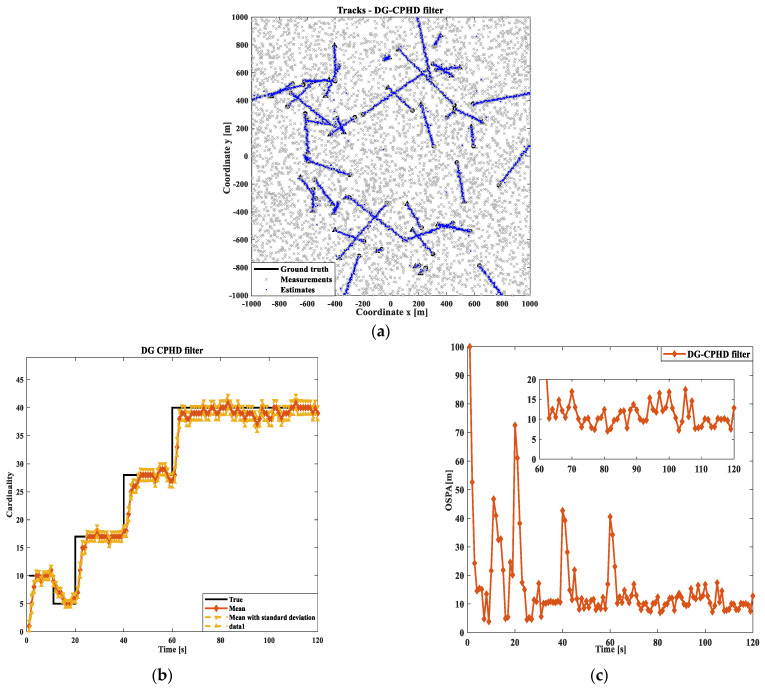
Comparison of GM-CPHD filtering results: (**a**) target tracking path result; (**b**) cardinal distribution results; (**c**) OSPA error results.

Firstly, the effects of the PHD, CPHD, and DG-CPHD filtering methods are compared, as shown in Figure 5, Figure 6 and Figure 7. At the same time, in order to avoid the potential impact of moving target motion trajectories on the detection results, this study uses randomly generated target motion trajectories to verify the tracking of multiple target motion results using different filtering methods. We conduct an investigation and analysis in terms of the following aspects. From the results of the three filtering methods shown in Figure 5, Figure 6 and Figure 7, it can be seen that the DG-CPHD filtering effect is similar to that of CPHD filtering. However, it is significantly higher than the PHD filtering effect, which means that in terms of the filtering effect, CPHD≈DGCPHD>PHD.

**Figure 8:**
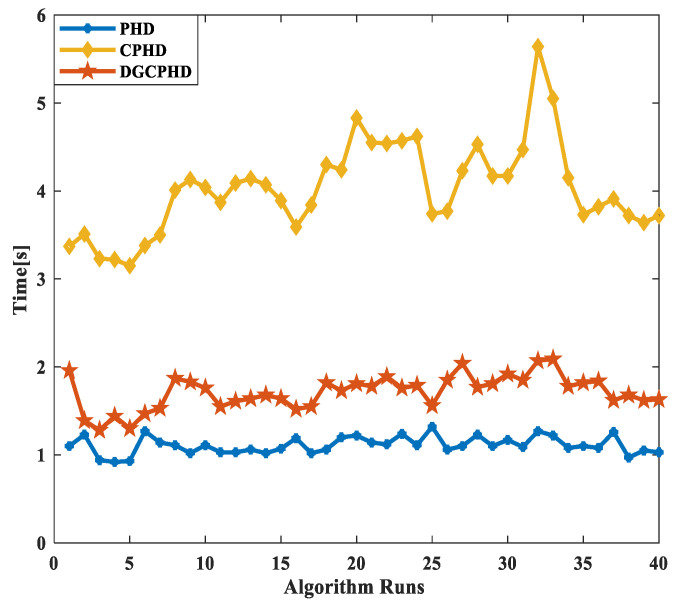
Comparison of running time results of three filtering methods.

Next, the running times of the PHD, CPHD, and DG-CPHD filtering methods are compared, and 40 algorithm runs are selected for comparison, as shown in Figure 8. The above comparison results suggest that the DG-CPHD filtering effect is similar to the CPHD filtering effect, but the calculation time can be significantly shorter than the CPHD filtering operation time. It can be seen that the DG-CPHD filter can be widely used in systems with high requirements for the calculation time and low requirements for the filtering effect. From the running time of the three filtering methods shown in Figure 8, CPHD has the longest filtering time, PHD has the shortest filtering time, and DG-CPHD has a running time between the two. In other words, from the perspective of the filtering effect, $t_{CPHD}>t_{DGCPHD}>t_{PHD}$.

**Figure 9:**
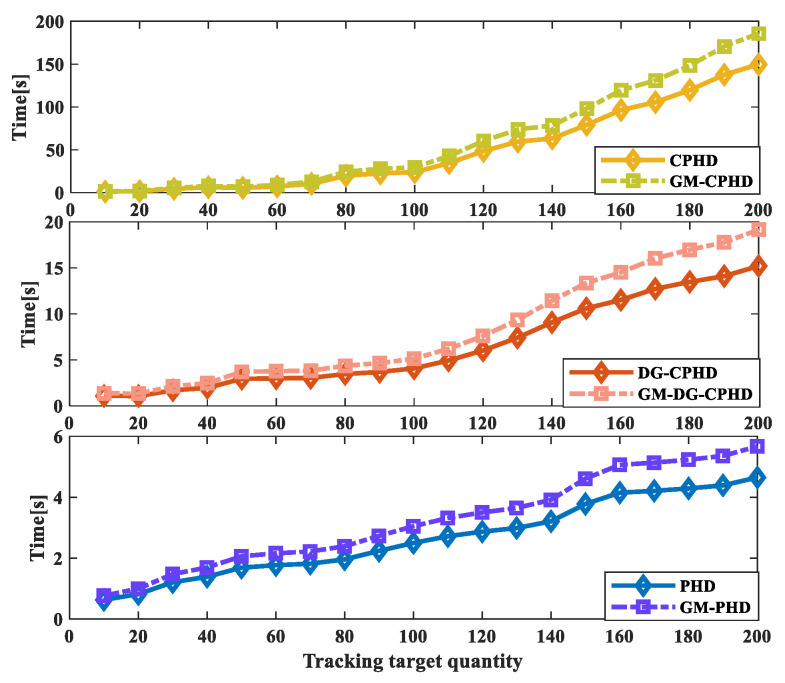
Three filtering methods used to track the running time results with different numbers of moving targets.

**Figure 10:**
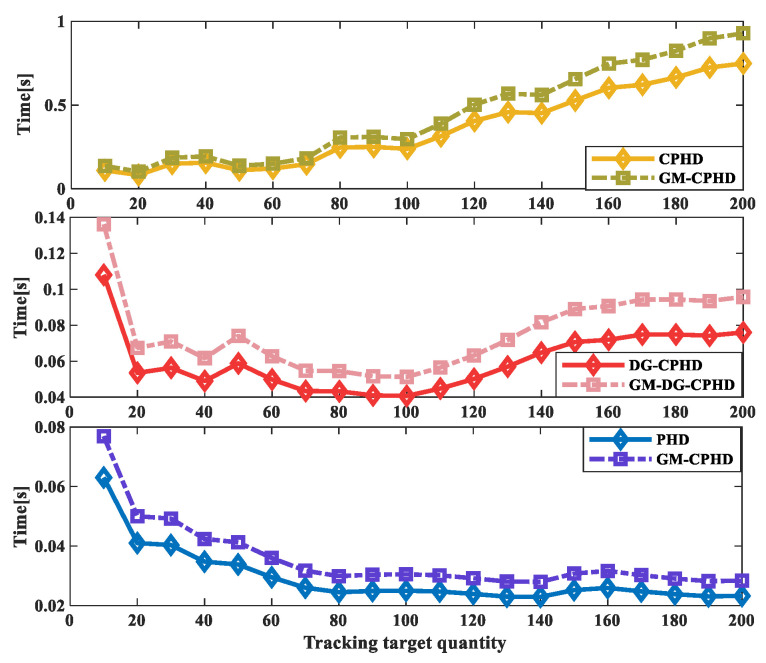
Results of the ratio of running time of three filtering methods to different numbers of moving targets.

The running time results when tracking different numbers of moving targets using the three filtering methods are compared, as shown in Figure 9. From the results, it can be seen that CPHD and GM-CPHD filtering increase the running time significantly with an increasing number of runs; PHD and GM-PHD filtering have a smaller increase in running time as the number of runs increases. The increase in the operating time of DG-CPHD is between the two. Thus, from the perspective of the effect of different runs over time, $t_{CPHD}^{\prime }>t_{DGCPHD}^{\prime }>t_{PHD}^{\prime }$. In order to further verify the effectiveness of different filtering methods with different numbers of runs over the running time, the ratio of the running time to the number of runs was calculated for the three filtering methods. From the results shown in Figure 10, it can be seen that the ratio of the running time of CPHD and GM-CPHD filtering to the tracking of different numbers of moving targets has a relatively large effect. The ratio of the PHD and GM-PHD filtering operation times to the operation frequency results is relatively small. The comparison of the ratio of the DG-CPHD filter’s running time to the number of runs is between the two. Thus, from the comparison results of the ratio of the filtering running time to the number of runs, it can be seen that $t_{CPHD}^{\prime \prime }>t_{DGCPHD}^{\prime \prime }>t_{PHD}^{\prime \prime }$.

From the above results, it can be seen that the effectiveness of DG-CPHD filtering and GM-DG-CPHD filtering is between that of PHD and CPHD filtering, but the running time can be between that of the other methods and is less affected by the number of runs. In other words, DG-CPHD filtering is suitable for systems that do not require high filtering performance but require a good operating time. For this purpose, DG-CPHD filtering and GM-DG-CPHD filtering are selected for research and analysis in this study.

### 4.2. Comparison Results of Multiple Fusion Methods

For comparative analysis, 20 linear Gaussian measurement models are selected. The initial state of the linear Gaussian measurement model target is shown in Table 1.

**Figure 11:**
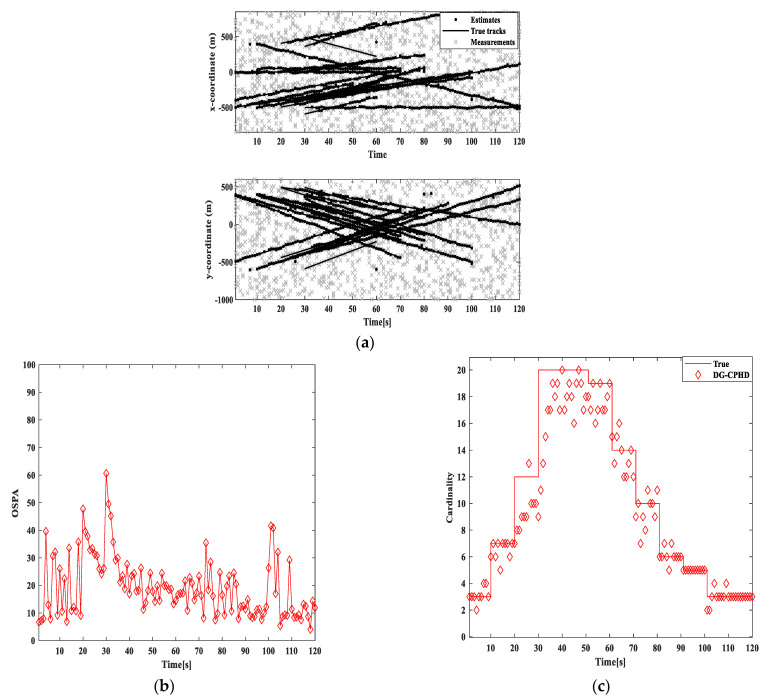
Comparison of GM-DG-CPHD filtering results: (**a**) target tracking path result; (**b**) cardinal distribution results; (**c**) OSPA error results.

**Figure 12:**
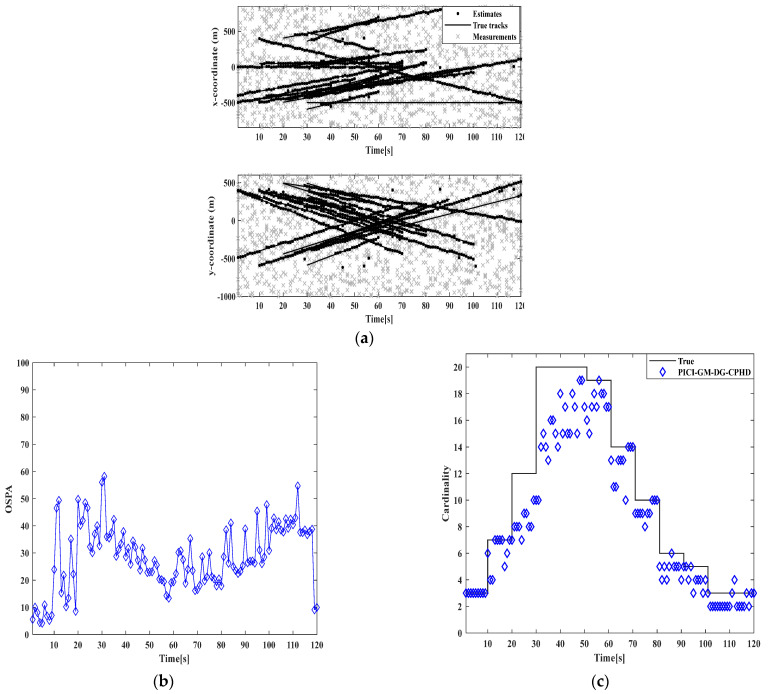
Comparison of PICI-GM-DG-CPHD filtering results: (**a**) target tracking path result; (**b**) cardinal distribution results; (**c**) OSPA error results.

Firstly, two algorithms, GM-DG-CPHD and PICI-GM-DG-CPHD, are implemented, as shown in Figure 11 and Figure 12. From the implementation effects of GM-DG-CPHD and PICI-GM-DG-CPHD in Figure 11 and Figure 12, it can be seen that the two algorithms can effectively achieve multi-objective motion tracking filtering for linear systems.

Secondly, the fusion effects of several methods, namely GM-DG-CPHD (single sensor 1), GICI-GM-DG-CPHD, SICI-GM-DG-CPHD, BICI-GM-DG-CPHD, and PICI-GM-DG-CPHD, are compared, as shown in the figures. From the 12 comparison results, it can be seen that the PICI-GM-DG-CPHD effect is second only to the GICI-GM-DG-CPHD effect. Compared with the SICI-GM-DG-CPHD and BICI-GM-DG-CPHD methods, it is more similar to the effect of GM-DG-CPHD (single sensor 1).

**Figure 13:**
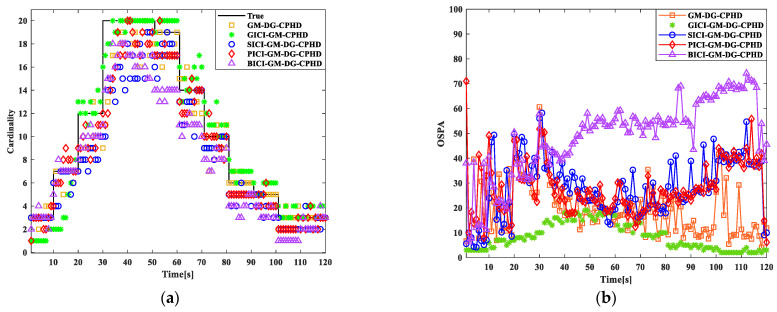
Comparison results of several filters: (**a**) cardinal distribution results; (**b**) OSPA error results.

**Figure 14:**
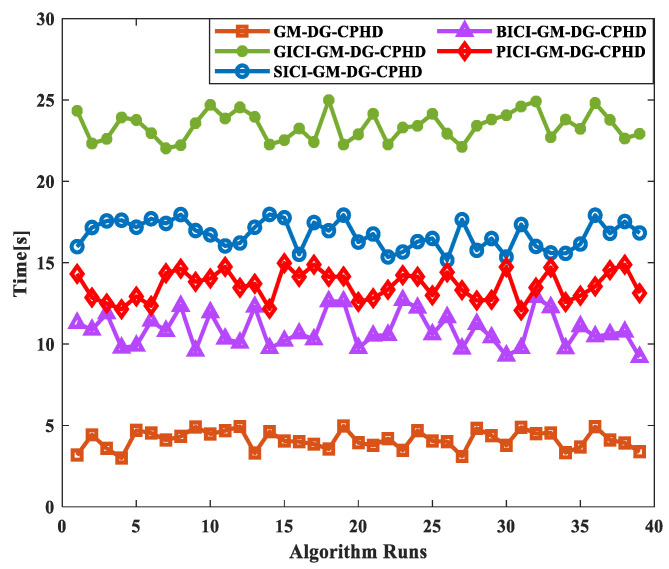
Comparison results of running times of several filters.

Finally, after verifying the comparison results of several filters’ effects, it is also necessary to verify the method running times of the fusion algorithms, as shown in Figure 13. We compare the running times of the GM-DG-CPHD (single sensor 1), GICI-GM-DG-CPHD, SICI-GM-DG-CPHD, BICI-GM-DG-CPHD, and PICI-GM-DG-CPHD methods, and we randomly select 40 algorithm runs for comparison, as shown in Figure 14. The above comparison results indicate that the filtering time of PICI-GM-DG-CPHD is between that of SICI-GM-DG-CPHD and BICI-GM-DG-CPHD, which is suitable for the efficient implementation of distributed sensor fusion.

### 4.3. Algorithm Complexity Analysis

Based on the research conclusion that the complexity of GM-DG-CPHD increases significantly with the increase in the number of targets, it can be seen that that of the PICI-GM-DG-CPHD algorithm also increases significantly with the increase in the number of targets, and it will be far greater than the recursive complexity $O(m^{3})∼O{\left|Z\right|}^{3}n_{\max})$ of CPHD. However, the running time of PICI-GM-DG-CPHD can be between that of SICI-GM-DG-CPHD and BICI-GM-DG-CPHD, which, to some extent, indicates that the computational complexity is between the two mentioned above.

## 5. Conclusions

This study proposes a GM-DG-CPHD filter based on PICI for the efficient implementation of distributed fusion in sensor networks and verifies the simulation performance through a linear Gaussian measurement model. Comparing PHD, GM-PHD, CPHD, GM-CPHD, DG-CPHD, and GM-DG-CPHD, the simulation results show that the DG-CPHD filtering effect is similar to the CPHD filtering effect, but the calculation time can be significantly shorter than the CPHD filtering running time, making it suitable for the efficient implementation of linear system target tracking. Comparing GM-DG-CPHD (single sensor 1), GICI-GM-DG-CPHD, SICI-GM-DG-CPHD, BICI-GM-DG-CPHD, PICI-GM-DG-CPHD, and PICI-GM-DG-CPHD, the simulation results show that PICI-GM-DG-CPHD can substantially reduce the computing time, and its performance is second only to that of GICI-GM-DG-CPHD, making it more suitable for distributed fusion with a large number of sensors.

## Figures and Tables

**Table 1:** Twenty linear Gaussian measurement models for the motion state of moving targets.

Target	Initial State	Appearing Frame	Disappearing Frame
1	[0; 0; 400; −12]	1	70
2	[−500; 5; 400; −9]	1	100
3	[−400; 6; −500; 10]	1	70
4	[400; −8; −600; 10]	10	120
5	[−500; 5; 400; −9]	10	70
6	[50; 0; 400; −10]	10	70
7	[−450; 7; 400; −6]	10	50
8	[−450; 7; −450; 10]	20	60
9	[400; 6; −500; 11]	20	90
10	[−500; 6; 500; −5]	20	120
11	[0; 4; 350; −8]	20	80
12	[−400; 4; 500; −10]	20	100
13	[−400; 9; −400; 12]	30	80
14	[350; 11; −400; 8]	30	60
15	[−600; 8; 500; −9]	30	60
16	[0; 5; 350; −12]	30	60
17	[−400; 5; 300; −10]	30	80
18	[−500; 0; −400; 8]	30	120
19	[500; −9; −600; 12]	30	60
20	[−450; 10; 400; −6]	30	80

## Data Availability

Data are contained within the article.
